# Bilirubin is produced nonenzymatically in plants to maintain chloroplast redox status

**DOI:** 10.1126/sciadv.adh4787

**Published:** 2023-06-07

**Authors:** Kazuya Ishikawa, Xiaonan Xie, Yasuhide Osaki, Atsushi Miyawaki, Keiji Numata, Yutaka Kodama

**Affiliations:** ^1^ Center for Bioscience Research and Education, Utsunomiya University, Tochigi 321-8505, Japan.; ^2^ Graduate School of Medicine, Dentistry, and Pharmaceutical Sciences, Okayama University, Okayama 700-8530, Japan.; ^3^ Laboratory for Cell Function Dynamics, RIKEN Center for Brain Science, Saitama 351-0198, Japan.; ^4^ Biotechnological Optics Research Team, RIKEN Center for Advanced Photonics; Saitama, 351-0198, Japan.; ^5^ Department of Material Chemistry, Graduate School of Engineering, Kyoto University; Kyoto, 615-8246, Japan.; ^6^ Biomacromolecules Research Team, RIKEN Center for Sustainable Resource Science, Saitama 351-0198, Japan.

## Abstract

Bilirubin, a potent antioxidant, is a product of heme catabolism in heterotrophs. Heterotrophs mitigate oxidative stress resulting from free heme by catabolism into bilirubin via biliverdin. Although plants also convert heme to biliverdin, they are generally thought to be incapable of producing bilirubin because they lack biliverdin reductase, the enzyme responsible for bilirubin biosynthesis in heterotrophs. Here, we demonstrate that bilirubin is produced in plant chloroplasts. Live-cell imaging using the bilirubin-dependent fluorescent protein UnaG revealed that bilirubin accumulated in chloroplasts. In vitro, bilirubin was produced nonenzymatically through a reaction between biliverdin and reduced form of nicotinamide adenine dinucleotide phosphate at concentrations comparable to those in chloroplasts. In addition, increased bilirubin production led to lower reactive oxygen species levels in chloroplasts. Our data refute the generally accepted pathway of heme degradation in plants and suggest that bilirubin contributes to the maintenance of redox status in chloroplasts.

## Teaser

Bilirubin, a typical heme degradation product in animals, is produced non-enzymatically in plants to maintain redox balance.

## INTRODUCTION

Bilirubin is a yellow pigment that is a product of heme catabolism in heterotrophs such as mammals. Heme is an essential biological molecule consisting of a tetrapyrrole ring and an iron cation. After being synthesized in mitochondria, heme is distributed throughout the cell and binds to various proteins as a cofactor, playing essential roles in cellular functions such as oxygen transport and electron transfer. However, free heme released from the breakdown of heme-containing proteins (hemoproteins) is a strong pro-oxidant that promotes cellular oxidative stress, and its levels are therefore tightly controlled via catabolism (*1*–*3*). Heme oxygenases open and oxidize the heme ring into the linear tetrapyrrole biliverdin, which is subsequently reduced to bilirubin by biliverdin reductase. Biliverdin reductase uses reduced form of nicotinamide adenine dinucleotide phosphate (NADP^+^) (NADPH) as an electron donor to reduce biliverdin (*4*), making the activity of biliverdin reductase NADPH dependent. Although bilirubin is toxic and causes kernicterus at high concentrations, it is also a potent antioxidant. Mildly elevated bilirubin levels in the body lower the risk of diseases such as arterial hypertension and diabetes mellitus by preventing cellular oxidative damage (*5*–*7*).

In plants, heme is biosynthesized and degraded in chloroplasts, unlike the typical mitochondrial biosynthetic pathway of heterotrophs. Aside from their different subcellular localizations, heme biosynthesis and degradation pathways are identical in plants and heterotrophs until the point of biliverdin biosynthesis via heme oxygenase. In plants, biliverdin is reduced to phytochromobilin by the plant-specific enzyme phytochromobilin synthase (*8*). Phytochromobilin is the chromophore of phytochromes, the red/far-red photoreceptors that promote photomorphogenesis and light-mediated development in plants. Phytochromobilin is believed to be the end product of heme degradation, since biliverdin reductase activity has not been detected in plant extracts (*9*, *10*). However, the sum of free and bound heme pools is estimated to far exceed the abundance of phytochromobilin in plants (*11*–*13*), suggesting the existence of other end products in the plant heme degradation pathway. Consistent with this idea, a few studies have reported the presence of bilirubin in the flowers and fruits of specific plant species, such as bird of paradise (*Strelitzia* spp.) (*14*, *15*).

Enzymes improve reaction rates and reaction specificities by catalyzing chemical reactions to control cellular functions. On the other hand, chemical reactions sometimes proceed in the absence of enzymes, with examples of nonenzymatic reactions playing important roles in cell metabolism (*16*, *17*). Several cases have been reported in which a reduced cofactor reacts nonenzymatically as an electron donor in vitro (*18*, *19*). However, the contribution of nonenzymatic reactions to cellular functions in vivo is largely unknown because genetic approaches are limited in their ability to investigate nonenzymatic reactions.

In this study, we used UnaG, a bilirubin-dependent fluorescent protein isolated from the muscle tissue of Japanese eel (*Anguilla japonica*), to demonstrate that bilirubin biosynthesis takes place in plants universally. UnaG has a specific and ultrahigh affinity for bilirubin (bilirubin IXα) and does not bind to biliverdin or any bilirubin analogs such as ditauro-bilirubin (*20*). Furthermore, we revealed that NADPH and biliverdin react nonenzymatically to produce bilirubin in chloroplasts, which have a considerably higher NADPH concentration than animal cells (*21*–*23*). Because increased bilirubin production diminished oxidative stress, we propose that nonenzymatically produced bilirubin contributes to the maintenance of redox status in chloroplasts.

## RESULTS

### Bilirubin production in plants

**Fig. 1.**
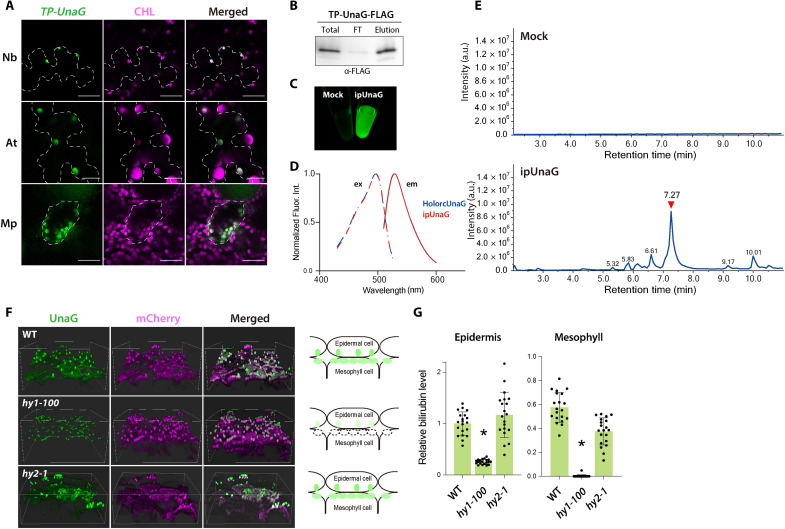
Bilirubin is synthesized in planta. (**A**) Transient expression of constructs encoding TP-UnaG after particle bombardment of *N. benthamiana* (Nb) and Arabidopsis (At) leaves and *M. polymorpha* (Mp) thalli. CHL, chlorophyll autofluorescence. Successfully transfected cells are outlined by dashed lines. Scale bars, 25 μm. (**B** to **E**) Immunopurification of holoUnaG from *N. benthamiana* leaves transiently expressing *TP-UnaG-FLAG*. (B) Immunoblot of immunopurified UnaG-FLAG (ipUnaG) using an anti-FLAG antibody. FT, flow-through fraction. (C) ipUnaG fluorescence under blue light excitation. A mock immunoprecipitated sample from nontransgenic leaves was processed in parallel. (D) Excitation (ex; dashed lines) and emission (em; solid lines) spectra of ipUnaG (red) and recombinant holoUnaG (holorcUnaG; blue). (E) Total ion chromatograms (TICs) of ipUnaG. The mock control is a sample immunoprecipitated from nontransgenic leaves. The red arrowhead indicates a peak with a product ion spectrum corresponding to bilirubin standards (fig. S1A). a.u., arbitrary unit. (**F** and **G**) *TP-mCherry-UnaG* expression in a mutant lacking either heme oxygenase 1 (*hy1-100*) or phytochromobilin synthase (*hy2-1*). (F) Three-dimensional (3D) visualization of UnaG and mCherry fluorescence in epidermal cells and mesophyll cells of 7-day-old seedlings from the wild-type (WT) and *hy1-100* and *hy2-1* mutants. The diagrams to the right illustrate side views of the 3D fluorescence images. Green, UnaG fluorescence. (G) Quantification of plastid UnaG fluorescence intensity in the WT and mutants. Intensity in the leaf epidermis of the WT was set to 1. Data are means ± SD (*n* = 20); asterisks indicate significant differences from the WT and *hy2-1*, *P* < 0.01, using Tukey’s multiple comparisons test.

We tested whether bilirubin accumulates in plants using the bilirubin biosensor UnaG. Since plant heme is biosynthesized and catabolized in chloroplasts, we transiently expressed a construct encoding a plastid (chloroplast)–targeted version of UnaG, with an N-terminal plastid transit peptide from Arabidopsis (*Arabidopsis thaliana*) Rubisco small subunit 1A (Rbcs1A) [transit peptide–fused UnaG (TP-UnaG)], in the leaves of *Nicotiana benthamiana*, Arabidopsis, and liverwort (*Marchantia polymorpha*). UnaG exhibited bright green fluorescence in the chloroplasts of all tested plants (Fig. 1A).

To ascertain whether UnaG is associated with endogenous bilirubin inside plant cells, we transiently expressed a construct encoding FLAG-tagged TP-UnaG (TP-UnaG-FLAG) in *N. benthamiana* leaves and immunopurified UnaG-FLAG using an anti-FLAG antibody. Immunoblot analysis demonstrated the efficiency of UnaG-FLAG immunoprecipitation (Fig. 1B). Immunopurified UnaG-FLAG (ipUnaG) emitted green fluorescence when excited with blue light (Fig. 1C). The excitation and emission spectra for ipUnaG and the control, holorcUnaG (recombinant UnaG complexed with commercial standard bilirubin), were identical (Fig. 1D). The perfect overlap in fluorescence spectra between ipUnaG and holorcUnaG suggests that UnaG-FLAG bound exclusively to bilirubin in plant cells.

To identify bilirubin bound to ipUnaG, we performed high-performance liquid chromatography (HPLC)–electrospray ionization mass spectrometry. A total ion chromatogram (TIC) of ipUnaG showed a major peak (Fig. 1E) with a tandem mass spectrum that was identical to that of standard bilirubin (fig. S1). Other peaks in Fig. 1E likely represent molecules derived from bilirubin isomers and fragmented ipUnaG. These results provide strong evidence that exogenously expressed UnaG specifically bound to bilirubin present in plant cells. Collectively, these data suggest that bilirubin is produced in a wide range of plant species and accumulates in chloroplasts.

Next, we expressed a construct encoding chloroplast-targeted mCherry-UnaG (TP-mCherry-UnaG) in Arabidopsis mutants lacking enzymes involved in heme degradation: heme oxygenase 1 (AtHO1) and phytochromobilin synthase (Fig. 1F). mCherry provided visual confirmation of UnaG expression in chloroplasts. In a mutant lacking AtHO1 (*hy1-100*, for *long hypocotyl 1*), which converts heme to biliverdin in wild-type (WT) plants (*24*), UnaG fluorescence was four times lower than in WT cotyledon epidermal cells (Fig. 1, F and G). In mesophyll cells, the fluorescence was almost undetectable in *hy1-100* (Fig. 1, F and G). In a mutant lacking phytochromobilin synthase (*hy2-1*), the enzyme that catalyzes the formation of phytochromobilin from biliverdin (*8*), UnaG fluorescence in both cotyledon epidermal cells and mesophyll cells was comparable to that in the WT (Fig. 1, F and G). These results suggest that the bilirubin production takes place after biliverdin but not after phytochromobilin biosynthesis.

### Biliverdin is nonenzymatically converted to bilirubin in the presence of higher concentrations of NADPH in vitro

**Fig. 2.**
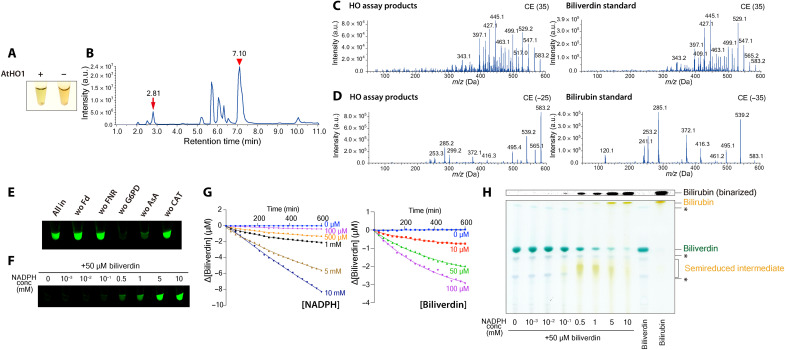
Bilirubin is converted from biliverdin nonenzymatically in the presence of high concentrations of NADPH. (**A** to **D**) In vitro heme oxygenase assay using recombinant AtHO1. (A) Reaction mixtures with recombinant AtHO1 (+AtHO1) or buffer (−AtHO1). (B) to (D) Mass spectrometry detection of biliverdin and bilirubin from the reaction products of the heme oxygenase assay. (B) TIC of the reaction products. The arrow at 2.81 min and the arrowhead at 7.10 min indicate peaks with a product ion mass spectrum matching that of the biliverdin standard (C) and bilirubin standard (D), respectively. (C) and (D) Product ion mass spectra derived from the precursor ion [M-H] + [mass/charge ratio (*m*/*z*) 583.1] (C) and [M-H] − (*m*/*z* 583.1) (D) were detected in positive or negative ion mode with the indicated collision energies (CEs) (left, reaction products; right, biliverdin and bilirubin standards). (**E** to **H**) Nonenzymatic production of bilirubin from biliverdin and NADPH. (E) Biliverdin was mixed with the materials required for heme oxygenase assay other than hemin and AtHO1 (all-in) or without the indicated materials [ferredoxin (Fd), ferredoxin-NADP^+^ reductase (FNR), G6PD, sodium ascorbate (AsA), and catalase (CAT)]. Bilirubin production was detected by the addition of recombinant apoUnaG. (F) Biliverdin (50 μM) was incubated with a series of concentrations of NADPH, and bilirubin production was detected by the addition of recombinant apoUnaG. (G) Time course of bilirubin production, measured at various concentrations of NADPH (with 50 μM biliverdin) or biliverdin (with 1 mM NADPH). (H) Thin-layer chromatography analysis of reaction products that were incubated with biliverdin and various concentrations of NADPH. Asterisks indicate biliverdin isomers. Top: A binarized image obtained by extracting yellow colors at the spot position of bilirubin IXα using Fiji software.

The heme oxygenase reaction in plants requires coordination between ferredoxin and other electron donors with heme oxygenase (*25*). Considering that homologs of biliverdin reductase genes have not been identified in plant genomes, we hypothesized that both biliverdin and bilirubin are produced by the action of ferredoxin and/or other electron donors during the heme degradation reaction. We thus performed an in vitro heme oxygenase assay that reconstitutes the natural internal environment of chloroplasts (*25*, *26*). This assay contains recombinant AtHO1, hemin (ferric heme), a ferredoxin-NADP^+^ oxidoreductase/ferredoxin electron transfer system, and an NADPH-regeneration system based on glucose-6 phosphate dehydrogenase (G6PD). After incubation at 25°C for 8 hours, the reaction solution with AtHO1 had a yellow-green color, whereas the buffer control remained the red color of heme (Fig. 2A). After stopping the reaction and removing the enzymes with organic solvents, we concentrated the reaction products and dissolved them in dimethyl sulfoxide (DMSO). Using liquid chromatography–mass spectrometry analysis, we determined whether bilirubin and biliverdin were synthesized. The TICs exhibited peaks with product ion mass spectra corresponding to bilirubin and biliverdin standards (Fig. 2, C and D), indicating the synthesis of bilirubin and biliverdin. These results suggest that bilirubin is produced during heme degradation despite the lack of biliverdin reductase.

We performed the following experiments to elucidate how bilirubin is produced in the in vitro heme oxygenase assay. Biliverdin was converted into bilirubin even in the absence of hemin and AtHO1 (Fig. 2E, lane: all-in). This observation indicated that enzymes and cofactors other than AtHO1 convert biliverdin to bilirubin. Therefore, we omitted additional enzymes and cofactors one by one to delineate the factors required for the conversion of biliverdin to bilirubin. We detected no bilirubin only when G6PD was omitted (Fig. 2E). G6PD is an enzyme that converts NADP^+^ to NADPH, and biliverdin is reduced to bilirubin using NADPH as an electron donor in heterotrophs. This led us to hypothesize that NADPH reduces biliverdin nonenzymatically to produce bilirubin. When mixing biliverdin and NADPH, we observed bilirubin production in an NADPH concentration–dependent manner (Fig. 2F). We thus reacted different concentrations of NADPH with biliverdin and monitored the decrease in biliverdin over time. Biliverdin was converted to bilirubin in NADPH and biliverdin concentration–dependent manners (Fig. 2G). The degradation rate of biliverdin appeared to be proportional to the two-thirds power of the concentrations of NADPH and biliverdin. When the reaction products of biliverdin and NADPH were separated by thin-layer chromatography, we detected two bilirubin-like yellow spots at high and low *R*_f_ values at the NADPH concentrations of 0.1 mM or higher (Fig. 2H, top). Spots with high *R*_f_ values were bilirubin, as they were consistent with the bilirubin standard. Yellow spots with low *R*_f_ values were predominantly produced at low NADPH concentrations and are thus presumed to be semireduced intermediates in which one hydrogen ion was introduced into the biliverdin molecule. Subsequently, another hydrogen ion would be introduced from NADPH to produce bilirubin. These results indicate that NADPH and biliverdin react nonenzymatically to produce bilirubin.

### Increased NADPH levels promote bilirubin production in planta

**Fig. 3.**
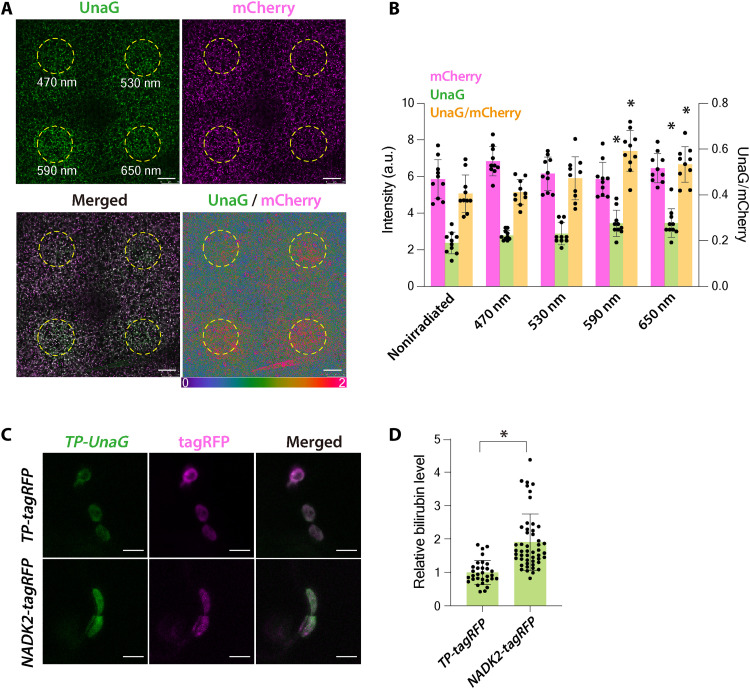
Increase in NADPH levels by *NADK2* overexpression or red light irradiation promotes bilirubin production. (**A** and **B**) High light irradiation increases bilirubin accumulation. (A) Eleven-day-old *TP-mCherry-UnaG*–expressing Arabidopsis true leaves were irradiated with a 470-, 530-, 590-, or 650-nm laser. The areas surrounded by the yellow dashed lines were irradiated intermittently for 2 hours. Scale bars, 50 μm. (B) Quantification of UnaG and mCherry fluorescence intensities and the UnaG/mCherry ratio in (A). Data are means ± SD (*n* = 10); *P < 0.01, using Dunnett’s multiple comparisons test. (**C** and **D**) Enhanced bilirubin accumulation upon the expression of Arabidopsis *NADK2* cloned in-frame with *tagRFP* (*NADK2-tagRFP*). (C) Confocal microscopy images of *N. benthamiana* cells transiently expressing *TP-UnaG* and *TP-tagRFP* or *NADK2-tagRFP*. Epidermal cells were observed 3 days after Agrobacterium-mediated infiltration. Scale bars, 5 μm. (D) Quantification of plastid bilirubin levels in *N. benthamiana* cells shown in (C). Plastid bilirubin levels in the cells coexpressing *TP-UnaG* and *TP-tagRFP* were set to 1. Data are means ± SD (*n* = 31 for *TP-mCherry-UnaG* and *n* = 49 for *TP-tagRFP*); *P < 0.01, using two-tailed Student’s *t* test.

High light exposure excites photosystem I and increases NADPH concentration in chloroplasts (*27*). If bilirubin is synthesized directly by reacting with NADPH, then bilirubin production would be expected to increase transiently during irradiation with high-intensity light. Therefore, we intermittently irradiated Arabidopsis leaves expressing *TP-mCherry-UnaG* with 470-, 530-, 590-, or 650-nm lasers for 2 hours by confocal laser scanning microscopy. Irradiation with 590- or 650-nm lasers significantly increased UnaG fluorescence and the UnaG/mCherry ratio (Fig. 3, A and B). The fluorescence intensity of mCherry was not attenuated, confirming that bleaching of the fluorescent protein did not occur. Wavelengths of 590 and 650 nm have higher relative quantum efficiencies for photosynthesis than 470 or 530 nm (*28*), suggesting that the photosystems were activated more strongly by these wavelengths and produced larger amounts of NADPH.

To further test for the nonenzymatic production of bilirubin in chloroplasts, we assessed TP-UnaG fluorescence in *N. benthamiana* cells overexpressing Arabidopsis *NAD KINASE 2* (*NADK2*). NADK2 phosphorylates NAD and increases the amount of NADP(H) in chloroplasts (*29*). We observed stronger UnaG fluorescence in the chloroplasts of cells expressing *NADK2-tagRFP* compared to those expressing *TP-tagRFP* [encoding red fluorescent protein (RFP) with a plastid transit peptide] (Fig. 3C). Quantification of UnaG fluorescence in chloroplasts revealed that *NADK2-tagRFP* overexpression significantly increases fluorescence intensity to twice that of the control (Fig. 3D), suggesting that increased NADPH levels promote bilirubin production in chloroplasts.

These results indicate that increasing NADPH concentration in chloroplasts promotes bilirubin production, supporting the idea that bilirubin production occurs in planta through the nonenzymatic reaction between biliverdin and NADPH.

### Bilirubin production lowers reactive oxygen species levels in chloroplasts

**Fig. 4.**
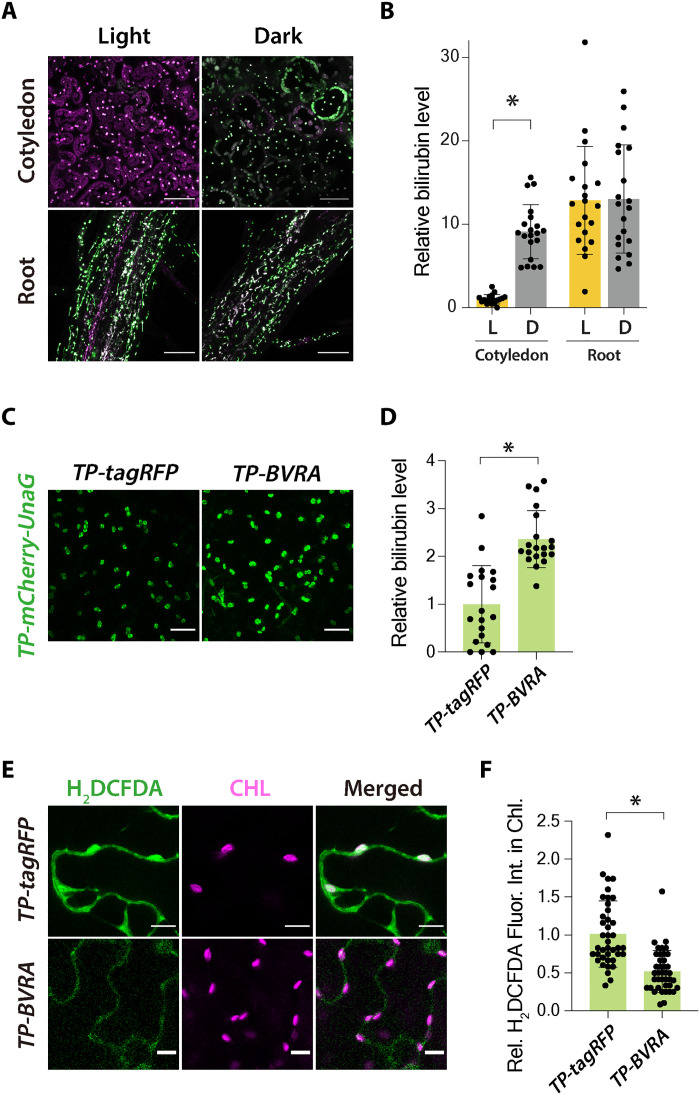
Bilirubin production decreases ROS accumulation in chloroplasts. (**A**) UnaG and mCherry fluorescence in cotyledons and the root elongation zone from 7-day-old Arabidopsis seedlings expressing *TP-mCherry-UnaG* exposed to light or darkness for 24 hours. Scale bars, 50 μm. (**B**) Quantification of plastid bilirubin levels in cotyledon mesophyll cells and root epidermal cells in (A). Plastid bilirubin levels in the cotyledons of light-grown seedlings were set to 1. L, light. D, dark. Data are means ± SD (*n* = 20); *P < 0.01, using two-tailed Student’s *t* test. (**C** to **F**) ROS accumulation in *N. benthamiana* leaves transiently expressing *TP-tagRFP* or *TP-BVRA*. (C) *TP-mCherry-UnaG* and *TP-tagRFP* or *TP-BVRA* were coexpressed in *N. benthamiana* leaves. Scale bars, 25 μm. (D) Quantification of chloroplast bilirubin levels. Data are means ± SD (*n* = 20). An asterisk indicates significant difference from the TP-tagRFP, *P < 0.01, two-tailed Student’s *t* test. (E) Epidermal cells stained with 50 μM of the ROS indicator H_2_DCFDA. Scale bars, 10 μm. (F) Quantification of H_2_DCFDA fluorescence intensity in chloroplasts. The mean value of *TP-tagRFP*–expressing leaves was set to 1. Data are means ± SD (*n* = 30); the asterisk indicates significant difference from *TP-tagRFP*, *P < 0.01, two-tailed Student’s *t* test.

We next explored the physiological functions of bilirubin in plants. High light irradiation stimulates bilirubin production (Fig. 3, C and D), but Arabidopsis seedlings grown in the dark for 24 hours showed increased bilirubin concentrations in leaves compared to light-grown seedlings (Fig. 4A). In mesophyll cells, chloroplast bilirubin levels were 9.1 times higher after 24 hours in the dark compared to those of light-grown seedlings (Fig. 4B). However, plastid bilirubin levels did not significantly change in root epidermal cells of dark-treated seedlings after 24 hours. As bilirubin has antioxidant properties in mammals (*5*–*7*), we hypothesized that, under moderate light conditions, bilirubin is degraded by reactive oxygen species (ROS) generated by photosynthesis faster than it is produced. To investigate the ROS scavenging activity of bilirubin, we examined ROS accumulation in *N. benthamiana* leaves in which chloroplast bilirubin accumulation was elevated by the heterologous expression of a plastid-targeted version of rat (*Rattus rattus*) biliverdin reductase A (*TP-BVRA*; fig. S2), an enzyme that catalyzes the reduction of biliverdin to bilirubin (*9*). *TP-BVRA* expression increased the chloroplast bilirubin pool relative to that seen in plants expressing *TP-tagRFP* (Fig. 4, C and D). Previous studies have shown that stable expression of *BVRA* affects photosynthesis (*9*, *10*), but this transient *BVRA* expression did not affect photosynthetic efficiency (fig. S3). To analyze ROS levels, we used the ROS indicator 2′,7′-dichlorodihydrofluorescein diacetate (H_2_DCFDA), which becomes fluorescent when it is oxidized in the presence of cellular ROS after the acetate groups are removed by intracellular esterases. Confocal microscopy revealed that TP-BVRA accumulation decreases H_2_DCFDA fluorescence in chloroplasts compared to control leaves expressing *TP-tagRFP* (Fig. 4E). Quantification of H_2_DCFDA fluorescence intensity in chloroplasts showed that the accumulation of TP-BVRA significantly lowers ROS levels (Fig. 4F), suggesting that the production of bilirubin diminishes chloroplast ROS levels. Therefore, we propose that bilirubin is constantly degraded in chloroplasts under light conditions by reacting with ROS generated by photosynthesis.

## DISCUSSION

**Fig. 5.**
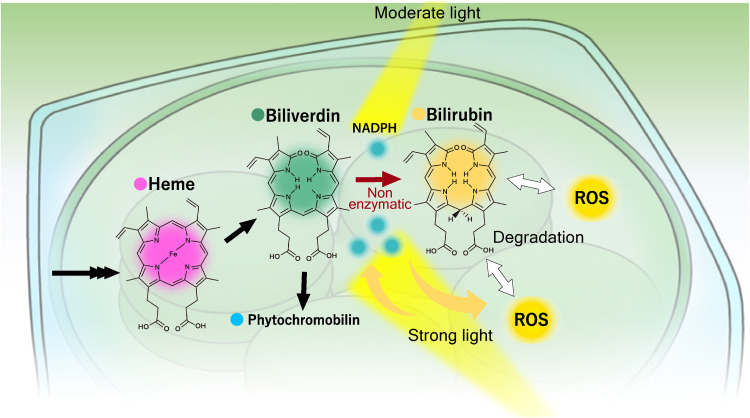
The bilirubin production pathway and its physiological functions. The conversion of heme to biliverdin is catalyzed by heme oxygenases, while the conversion of biliverdin to phytochromobilin is catalyzed by phytochromobilin synthase (black arrows). Biliverdin reacts nonenzymatically with NADPH and is converted to bilirubin (brown arrows). An excess of NADPH is produced upon the exposure to high-intensity light, and ROS and biliverdin production are accelerated (yellow arrows). Bilirubin may contribute to the maintenance of the redox status in chloroplasts by reacting with excessive NADPH generated under high light and by reacting with ROS generated by photosynthesis (white arrows).

This study provides evidence that bilirubin, believed to be an animal pigment, is produced from biliverdin in plants nonenzymatically. Since we detected bilirubin in the early-diverging land plant *M. polymorpha* as well as the angiosperms *N. benthamiana* and Arabidopsis (Fig. 1A), we propose that almost all land plants, including crops, probably produce bilirubin. Our findings also revise the generally accepted pathway for heme degradation of plants (Fig. 5).

The NADPH-mediated nonenzymatic conversion of biliverdin described here appears to be kinetically slower than the biliverdin reductase–mediated reaction in mammals. Considering the physiological differences between animals and plants, we posit two potential reasons for the nonenzymatic bilirubin production in plants. First, plants catabolize much less heme than animals. In adult humans, 375 mg of heme, which corresponds to approximately 10 nmol/g, is degraded each day (*16*), mainly due to hemoglobin turnover associated with oxygen transport in the blood. By contrast, the unbound heme content in aerial tissues of 3-week-old Arabidopsis plants was estimated to be 0.75 nmol/g of fresh weight (*11*). Even if all heme were degraded in 1 day, the amount of degraded heme by body mass in plants would be approximately ^1^/_10_ that of humans. Therefore, plants may simply not need a high-speed heme degradation system comparable to that of animals. Second, NADPH concentrations in plant cells are higher than in animal cells. The NADPH concentration in animal cells is estimated to be 3 μM in the cytoplasm and 37 μM in mitochondria (*21*). By contrast, NADPH concentrations in the chloroplast stroma are estimated to be 0.29 to 0.5 mM (*22*, *23*). This high NADPH concentration probably enables nonenzymatic production of bilirubin with a relatively high reaction rate. On the basis of our results, we estimate that biliverdin is degraded at approximately 0.27 μM/hour when 50 μM biliverdin is incubated with 0.5 mM NADPH (Fig. 2G). In human cell cultures, the rate of bilirubin production, which is primarily mediated by BVRA, is estimated to be a few micromolars per hour (*30*), indicating that the estimated bilirubin production rate in plant cells is not much slower than that in mammal cells. This reaction rate would be further increased if NADPH and biliverdin were concentrated in subdomains within the chloroplast. Ferredoxin transfers electrons generated by photosystem I to NADP^+^, resulting in NADPH production, but ferredoxin is also required as an electron donor for heme oxygenase activity (*25*). The production of biliverdin and NADPH therefore increases simultaneously when photosystem I is activated. On the thylakoid membrane, where photosystem I is located, higher concentrations of biliverdin and NADPH than those mentioned above may occur locally, resulting in higher bilirubin production rates than our estimation.

We propose that nonenzymatic bilirubin production in plants temporarily stores and mitigates excess reducing power (Fig. 5). Plants contain photosystem I, a system that produces NADPH using absorbed light energy, since they consume a large amount of NADPH for CO_2_ assimilation and photorespiration under light conditions. However, when exposed to high-intensity light, an excess of NADPH is produced and the electrons that can no longer be received by NADP^+^ are transferred to oxygen instead, causing the generation of ROS. Biliverdin may act as a reservoir of excess reducing power, attenuating photosystem I–mediated increases in NADPH levels while producing bilirubin as a potent antioxidant. This idea is consistent with the transient increase in bilirubin accumulation seen upon exposure to high-intensity light (Fig. 3, A and B). Bilirubin accumulated even in the dark, when photosynthesis is stopped and chloroplast NADPH consumption is low (Fig. 4, A and B). This bilirubin is likely derived from NADPH synthesized from the pentose phosphorylation pathway and may serve as a reservoir of reducing power for the regulation of ROS levels during photosynthesis. The ROS scavenging function of bilirubin in plant cells was suggested by the lower chloroplast ROS levels observed when bilirubin production was promoted by ectopic *BVRA* expression (Fig. 4, C to F). The bilirubin level calculated based on UnaG fluorescence intensity approximately doubled upon *BVRA* expression, while the ROS level was approximately halved, suggesting that bilirubin is a potent antioxidant. This notion is compatible with the removal of a 10,000-fold molar excess of H_2_O_2_ by bilirubin in human cells (*31*, *32*). However, we cannot conclude that the decrease in ROS levels is solely due to the effect of bilirubin because the animal enzyme BVRA was ectopically expressed in chloroplasts. One concern is that BVRA functions as a transcription factor in addition to bilirubin production, as reported previously for animal cells (*33*). Although BVRA is unlikely to function as a transcription factor in the present study because BVRA was targeted to the chloroplasts, the possibility of exhibiting side effects, such as binding to the chloroplast genome, cannot be denied. Furthermore, our results seem to be inconsistent with several papers suggesting that biliverdin, which is produced as a result of increased heme oxygenase expression, plays a protective role against oxidative stress in plants (*34*–*37*). The nonenzymatic production of bilirubin revealed in the present study points out that the antioxidative activity seen in the previous studies may include the effect of bilirubin. On the other hand, biliverdin also exhibits antioxidant activity in animal cells, although at a lower level than that of bilirubin (*38*). Increased heme oxygenase expression may provide resistance to oxidative stress through the combined antioxidant effects of biliverdin and bilirubin.

Recent studies revealed that bilirubin functions in signal transduction and regulation of metabolism as a hormone-like substance in animals (*39*, *40*). Similarly, in plants, the heme degradation pathway involves retrograde signaling from the chloroplast to the nucleus (*41*). Heme and bilin pigments produced by the heme degradation are generally thought to regulate photosynthetic gene expression as signaling molecules (*42*, *43*). Furthermore, ROS produced in photosynthesis also function as retrograde signaling molecules (*44*–*46*). Bilirubin and/or bilirubin-regulated ROS may function as retrograde signaling molecules or have hormone-like effects in plant cells similar to those seen in animals.

Cells contain a mixture of chemical substances, including unstable molecules, and nonenzymatic reactions are thought to occur everywhere. Furthermore, plants grow in an environment exposed to fluctuating light, whose energy frequently induces nonenzymatic reactions. Gene expression changes, which take tens of minutes to several hours, are not sufficient to respond to the constantly changing light environment. Plants may use a variety of nonenzymatic reactions to buffer the effect of fluctuating environments. However, there are few cases in which the physiological roles of these nonenzymatic reactions have been elucidated due to the difficulty of genetic analysis and in vivo experiments. Using a bilirubin biosensor called UnaG, we found that bilirubin is produced nonenzymatically in plants and maintains the redox status of chloroplasts. Further research with new perspectives and approaches is needed to elucidate additional physiologically important nonenzymatic reactions that have yet to be found in plants.

## MATERIALS AND METHODS

### Plant materials and growth conditions

We used Arabidopsis (*A. thaliana*) accession Columbia-0 (Col-0; CS60000) as the WT. Mutant lines were obtained from the Arabidopsis Biological Resource Center (*hy1-100*, CS236; *hy2-1*, CS2068). Arabidopsis seeds were surface sterilized and sown on Murashige and Skoog (MS) medium with 0.5% (w/v) gellan gum and 1% (w/v) sucrose. Arabidopsis seedlings were grown under continuous light (fluorescent tubes, 25 μmol m^−2^ s^−1^) at 22°C and were transferred to vermiculite 11 days after sowing to set seeds. *N. benthamiana* plants were grown under a 16-hour light/8-hour dark photoperiod at 25°C. *M. polymorpha* plants (Takaragaike-1) were grown as previously reported (*47*).

### Plasmid construction and transformation

All primers used for plasmid construction are listed in table S1. Unless otherwise noted, polymerase chain reaction (PCR)–amplified DNA fragments and a synthetic DNA fragment were cloned into the pENTR1a vector (Invitrogen) at the Sal I and Eco RV sites using the In-Fusion HD Cloning Kit (Clontech) according to the manufacturer’s instructions. For the cloning of *TP-mCherry-UnaG*, DNA fragments that encode the N-terminal transit peptide (79 amino acids) from Rbcs1A (*48*) were amplified by PCR using Col-0 genomic DNA as a template. Genomic DNA was extracted from Col-0 with the DNeasy Plant Mini Kit (QIAGEN) according to the manufacturer’s instructions. The PCR products were joined with the fragments encoding mCherry-UnaG, which was amplified from pcDNA3-mCherry-UnaG, by recombinant PCR. For the cloning of *TP-UnaG*, a linear pENTR1A-TP-UnaG fragment was amplified by inverse PCR using pENTR1A-TP-mCherry-UnaG as a template and circularized using the In-Fusion HD Cloning Kit. The *TP-UnaG-FLAG* fragment was amplified from pENTR1A-TP-UnaG. The synthetic DNA fragment encoding TP-BVRA was designed according to a previous study (*49*) and prepared as a synthetic double-stranded DNA (Integrated DNA Technologies; fig. S3). For the cloning of *NADK2*, the full-length coding sequence of *NADK2* (*29*) was amplified by PCR using Col-0 cDNA as a template. For the cloning of *TP-tagRFP*, the fragment encoding the N-terminal transit peptide of Rbcs1A and the *tagRFP* fragment amplified from the pGWB459 vector (*50*) were joined by recombinant PCR. The *TP-mCherry-UnaG* fragments cloned in pENTR1A were recombined into the pGWB602 binary vector, which was designed to express the cloned genes under the control of the cauliflower mosaic virus 35*S* promoter (*50*), using the Gateway LR reaction (Invitrogen). Transgenic Arabidopsis plants were generated using the floral dip method (*51*). The *TP-UnaG-FLAG*, *TP-BVRA*, and *TP-tagRFP* fragments were transferred to the pGWB511 binary vector (*50*), which expresses the cloned genes as a C-terminal FLAG-tagged fusion protein driven by the 35*S* promoter. The *NADK2* fragment was transferred to the pGWB560 binary vector (*50*), which expresses the cloned genes as a C-terminal tagRFP-tagged fusion protein driven by the 35*S* promoter. For particle bombardment, the *TP-UnaG* fragment was transferred to the pGWT35S vector, which places the cloned gene under the control of the 35*S* promoter (*52*), using the Gateway LR reaction. For the preparation of recombinant protein, *UnaG* fragments were amplified from pcDNA3-mCherry-UnaG and cloned into pDONR207 using the Gateway BP reaction (Invitrogen). A DNA fragment encoding amino acids 51 to 231 of AtHO1 was amplified from Arabidopsis cDNA (*26*) and cloned into the pENTR1a vector. Total RNA extraction and reverse transcription were performed according to a previous study (*53*). The *UnaG* and *AtHO1* fragments were transferred to the pDEST17 vector (Invitrogen), which is designed to express the cloned genes as a 6×His-tag fusion protein in *Escherichia coli*, using the LR reaction.

### Transient expression by particle bombardment and agroinfiltration

pGWT35S-TP-UnaG was delivered into *N. benthamiana*, Arabidopsis, and *M. polymorpha* by particle bombardment according to previously published protocols with minor modifications (*54*). True leaves of 4-week-old *N. benthamiana* plants or 11-day-old Arabidopsis seedlings were placed on top of plates containing 0.4% (w/v) agarose and bombarded using 650 and 900 psi of rupture discs, respectively. One-day-old gemmalings of *M. polymorpha* growing on the medium were bombarded using 900 psi of rupture discs (*55*). All samples were visualized by confocal microscopy 24 hours after bombardment. For agroinfiltration, Agrobacterium (*Rhizobium radiobacter*) strain GV2260 harboring pGWB602-TP-UnaG, pGWB602-TP-mCherry-UnaG, pGWB560-NADK2, pGWB511-TP-UnaG-FLAG, pGWB511-TP-BVRA, or pGWB511-TP-tagRFP was cultured, and the cells were collected by centrifugation and resuspended in pure water to a final optical density of 1.0 at 600 nm. Single or mixed Agrobacterium cultures were infiltrated into true leaves of 4-week-old *N. benthamiana*. The leaves were used for experiments 2 or 3 days after infiltration.

### Immunopurification of holoUnaG from plant cells

About 1 g of *N. benthamiana* leaves infiltrated with pure water or Agrobacterium cells harboring pGWB511-TP-UnaG-FLAG was collected. All subsequent procedures were performed at 4°C. The leaves were ground in a mortar and pestle with 5 ml of 1× phosphate-buffered saline (PBS; pH 7.4). The homogenate was filtered through two layers of Miracloth (Merck) and centrifuged at 3000*g* for 10 min. The supernatant was mixed with 100 μl of anti-DYKDDDDK (FLAG) tag antibody beads (Wako) and rotated for 5 hours. The mixture was transferred to a Poly-Prep Chromatography Column (Bio-Rad) to trap the affinity beads, which were subsequently washed twice with 5 ml of 1× PBS. The affinity beads were resuspended in 150 μl of 1× PBS, and UnaG florescence was observed under blue light. Further analysis was performed with UnaG bound to the affinity beads. Immunoblot analysis was carried out as described previously using an anti-DYKDDDDK (FLAG) antibody (Wako) and an anti-mouse antibody (Thermo Fisher Scientific) (*56*). Fluorescence excitation and emission spectra of ipUnaG were acquired using an F-2700 fluorescence spectrophotometer (Hitachi).

### Recombinant protein preparation

The recombinant proteins 6×His-tagged UnaG and AtHO1 were purified using the QIAexpress Ni-NTA Protein Purification System (QIAGEN). The pDEST17-UnaG and pDEST17-AtHO1 plasmids were transformed into *E. coli* BL21-AI (Invitrogen). The *E. coli* cells harboring each plasmid were cultured in LB medium at 37°C to an optical density at 600 nm of 0.4, and the production of UnaG was induced by the addition of 20% (w/v) d-(−)-arabinose (final concentration of 0.1%, w/v). After cultivation at 16°C for 24 hours, the cells were pelleted and resuspended in 1× tris-buffered saline (TBS) [20 mM tris-HCl (pH 7.9) and 500 mM NaCl]. The resuspended cells were sonicated on ice (SONIFIER 250, Branson) and centrifuged at 12,000*g* at 4°C for 15 min. Only when purifying recombinant holoUnaG (holorcUnaG) was a solution of bilirubin (Wako) added to the supernatant to a final concentration of 100 μM for holoUnaG formation. The supernatant was mixed with Ni-NTA Agarose (QIAGEN) and rotated at 4°C for 30 min. The mixture was transferred to a Poly-Prep Chromatography Column to trap the agarose resin and subsequently washed twice with five bed volumes of 1× TBS. The recombinant proteins were eluted from the resin by adding elution buffer [20 mM tris-HCl (pH 7.9), 500 mM NaCl, and 1 M imidazole] and buffer exchanged by PD-10 gel filtration column (Cytiva) for further experiments [1× PBS for apoUnaG and holorcUnaG or 100 mM potassium phosphate buffer (pH 7.2) for AtHO1]. Fluorescence excitation and emission spectra of holorcUnaG were acquired using an F-2700 fluorescence spectrophotometer (Hitachi).

### In vitro heme oxygenase assay

An in vitro heme oxygenase assay was performed according to a previous study (*25*, *26*). The following reagents were mixed and incubated at 25°C for 8 hours in a final volume of 100 μl: 5 μM recombinant AtHO1, 40 μM hemin (Tokyo Chemical Industry, catalog no. H0008), bovine serum albumin (60 μg/ml; Merck, catalog no. A9647), 4.6 μM ferredoxin (Merck, catalog no. F5875), ferredoxin-NADP^+^ reductase (0.025 U/ml; Merck, catalog no. F0628), 10 μM catalase (Merck, catalog no. 219261), 6.5 mM glucose-6-phosphate (Merck, catalog no. 10127647001), G6PD (Merck, catalog no. G7877), 0.82 mM NADP^+^ (Wako, catalog no. 308-50463), and 5 mM sodium ascorbate (Tokyo Chemical Industry, catalog no. A0539). These reagents were dissolved in 100 mM potassium phosphate buffer (pH 7.2) or DMSO according to the previous study (*26*). The reactions were stopped and their proteins removed by mixing water (reaction solution), methanol, and chloroform to a 0.9:1:1 ratio. Following centrifugation at 13,000*g* for 5 min, the upper layer was collected, dried, and then dissolved in 10 μl of DMSO. In Fig. 2E, biliverdin was added in place of AtHO1, hemin, and the indicated enzymes or coenzyme. The reaction products were mixed with an equal volume of 10 μM apoUnaG, and UnaG florescence was observed under blue light.

### Nonenzymatic reaction of biliverdin and NADPH in vitro

Biliverdin (Toronto Research Chemicals, catalog no. B386400) and NADPH (Wako Chemicals, catalog no. 44332000) were dissolved in DMSO and 100 mM potassium phosphate buffer (pH 7.2) and then mixed in 100 mM potassium phosphate buffer (pH 7.2) at 25°C. After 8 hour incubation, the reaction products were mixed with an equal volume of 10 μM apoUnaG, and UnaG florescence was observed under blue light. Biliverdin degradation was monitored by absorbance at 620 nm with a Multiskan FC Microplate Reader (Thermo Fisher Scientific) at 25°C. The reaction product (250 μl) after 24-hour incubation was lyophilized and dissolved in 15 μl of chloroform:methanol (1:1). The bilirubin and biliverdin standards were diluted in chloroform:methanol (1:1). The samples and 10 nmol of commercial biliverdin and bilirubin were loaded onto Merck 60 F254-coated silica gel plates (0.25 mm in thickness) and separated in chloroform:methanol:water (65:25:3).

### Confocal microscopy

Live-cell fluorescence imaging was performed with an SP8X confocal microscope system (Leica Microsystems) equipped with Fluotar VISIR 25× and HC PL APO CS 63× water-immersion lenses. Plant samples were mounted onto glass slides in pure water and topped with a coverslip. All images were taken in photon counting mode with the time-gating method (*57*). UnaG, mCherry, and H_2_DCFDA were excited with 498-, 554-, and 484-nm lasers from the white light laser source and detected at 510 to 545, 565 to 636, and 494 to 545 nm by hybrid detectors, respectively. Chlorophyll autofluorescence was detected at 655 to 743 nm by a photomultiplier tube detector. Images were taken at 100 Hz/1024 × 1024 pixels in sequential scan mode. The *z*-projection images of Figs. 3A and 4A were reconstructed from *z*-stack images captured in 3.5-μm intervals. Three-dimensional (3D) images of Fig. 1F were reconstructed from *z*-stack images taken at 100 Hz/256 × 256 pixels in 0.35-μm intervals using 3D viewer in Leica Application Suite X software. Lasers were irradiated using a sequential scan between frames. In each frame, lasers of 470, 530, 590, or 650 nm with 15% intensity from the 70% power white light laser source were irradiated to the regions of interest. The lasers of four different wavelengths were irradiated at 400 Hz/512 × 512 pixels (1.295 s per frame) in turn for 2 hours.

### Measurement of bilirubin levels

For the quantification of bilirubin levels or UnaG and mCherry fluorescence intensities, confocal images of UnaG and mCherry were taken at 100 Hz/1024 × 1024 pixels with the Fluotar VISIR 25× water-immersion lens. Each circular region of interest with 5 × 5 pixels was randomly set on the target plastids in different cells, and fluorescence intensities of UnaG and mCherry were measured with Fiji software (*58*). Bilirubin levels in *N. benthamiana* leaf epidermal cells were calculated as the mean value of UnaG fluorescence intensity. Bilirubin levels in epidermal cells of Arabidopsis were calculated with the following formula

$$\text{bilirubin level}=\frac{1}{n}\;\sum_{i=1}^{n}\left(x_{i}\frac{\bar{y}}{y_{i}}\right)$$

where *x* is UnaG fluorescence intensity, *y* is mCherry fluorescence intensity, and $\bar{y}$ is average mCherry fluorescence intensity.

For mesophyll cells, the following formula was used to normalize the fluorescence attenuation due to sample thickness

$$\text{bilirubin levels}=\frac{{\bar{y}}_{\mathrm{epi}}}{\bar{y}}\;\left[\frac{1}{n}\;\sum_{i=1}^{n}\left(x_{i}\frac{\bar{y}}{y_{i}}\right)\right]=\frac{1}{n}\;\sum_{i=1}^{n}\left(x_{i}\frac{{\bar{y}}_{\mathrm{epi}}}{y_{i}}\right)$$

where ${\bar{y}}_{\mathrm{epi}}$ is average mCherry fluorescence intensity in the epidermis of cotyledons.

### Investigation of the antioxidant function of bilirubin in planta

*N. benthamiana* leaves from the same position along the main stem were infiltrated with Agrobacterium cells harboring pGWB511-TP-BVRA or pGWB511-TP-tagRFP and analyzed 2 days after infiltration. The quantum yield photochemistry of photosystem II (*F*_v_/*F*_m_) was measured with a JUNIOR-PAM chlorophyll fluorometer (Walz). Measurements were made on the Agrobacterium-infiltrated leaves of three individual plants. The *F*_v_/*F*_m_ values were measured at three points on a leaf, and the average value was used as single-sample data. Three leaf discs (5 mm in diameter) that were taken from the Agrobacterium-infiltrated leaves in three plants were immersed in 50 μM H_2_DCFDA (Invitrogen) for 30 min by pressure infiltration with a syringe and subsequently washed with pure water. Fluorescence images were taken with a Leica SP8X as described above. H_2_DCFDA fluorescence intensities in chloroplasts were measured with Fiji software.

### Mass spectrometry analysis

Mass spectrometry analysis of proton adduct ions was performed with a triple quadrupole/linear ion trap instrument (LIT) (QTRAP5500, AB Sciex) with an electrospray source. MS and MS2 spectra were recorded in product ion scan mode using LIT. Ion source was maintained at 450°C with curtain gas at 30 psi, collisionally activated dissociation gas at 9 psi (12 psi for LIT), ion source gas at 80 psi, and ion source gas 2 at 70 psi. Ion spray voltage was set to 5000 V in positive ion mode and − 3500 V in negative ion mode. Declustering, entrance, and collision cell exit potentials were maintained at 60, 15, and 9 V, respectively. HPLC separation was performed on an ultrahigh-performance liquid chromatograph (Nexera X2, Shimadzu) equipped with a C8 column (YMC-Triart C8, ϕ 2.1 × 150 mm, 1.9 μm; YMC). The column oven temperature was maintained at 40°C. The mobile phase consisted of acetonitrile (solvent A) and water (solvent B), both of which contained 0.1% (v/v) acetic acid. HPLC separation was conducted with a linear gradient of 10% A (0 min) to 45% A (15 min) at a flow rate of 0.2 ml/min.
