# Supplementary material for: Bilirubin is produced nonenzymatically in plants to maintain chloroplast redox status
Source: Sci Adv. 2023 Jun 7;9(23):eadh4787. doi: 10.1126/sciadv.adh4787 (PMC10246902; doi:10.1126/sciadv.adh4787)
Supplement: Supplementary file 1 — Figs. S1 to S3 Table S1 [file sciadv.adh4787_sm.pdf]

Science Advances
AAAS

Supplementary Materials for

Bilirubin is produced nonenzymatically in plants to maintain chloroplast redox status

Kazuya Ishikawa *et al.*

Corresponding author: Yutaka Kodama, kodama@cc.utsunomiya-u.ac.jp

Sci. Adv. **9**, eadh4787 (2023)
DOI: 10.1126/sciadv.adh4787

This PDF file includes:

Figs. S1 to S3
Table S1

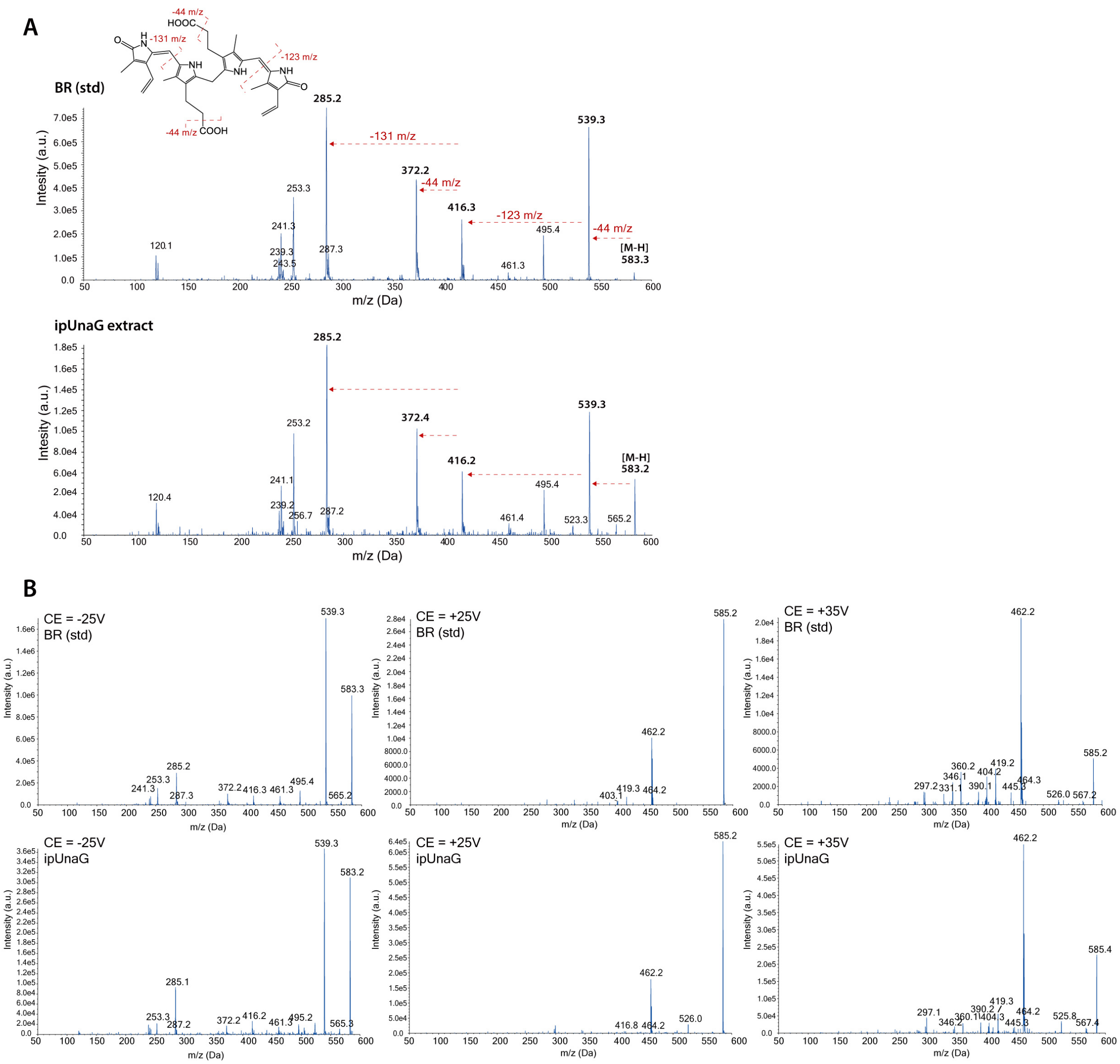

Fig. S1. Electrospray ionization–mass spectrometry analysis of ipUnaG. (**A**) Electrospray ionization–mass spectrometry of ipUnaG and bilirubin standards. Samples were negatively ionized with 35 V collision energy. The extracts of ipUnaG and authentic bilirubin standard were analyzed by liquid chromatography–tandem mass spectrometry (LC-MS/MS). The MS/MS spectra of the bilirubin standard and ipUnaG using the product ion scan mode are shown. Major fragmentation peaks are marked in red. a.u., arbitrary unit. (**B**) MS/MS data detected in negative and positive ion modes with each collision energy at –25, 25, or 35 V. The product ion mass spectra derived from the precursor ion [M–H]– (m/z 583.1) and [M+H]+ (m/z 585.1) of bilirubin standard and ipUnaG are shown. a.u., arbitrary unit.

```
>TP-BVRA

AACCAATTCAGTCGACatggcttcctctatgctctcttccgctactatggttgcctctccggctcaggcc
                M  A  S  S  M  L  S  S  A  T  M  V  A  S  P  A  Q  A
actatggtcgctcctttcaacggacttaagtcctccgctgccttcccagccacccgcaaggctaacaac
T  M  V  A  P  F  N  G  L  K  S  S  A  A  F  P  A  T  R  K  A  N  N
gacattacttccatcacaagcaacggcggaagagttaactgcatgcaggtgtggcctccgattggaaag
D  I  T  S  I  T  S  N  G  G  R  V  N  C  M  Q  V  W  P  P  I  G  K
aagaagtttgagactctctcttaccttcctgaccttaccGGCGGTAGCGGCGGTatggatgccgagcca
K  K  F  E  T  L  S  Y  L  P  D  L  T  G  G  S  G  G  M  D  A  E  P
aagaggaaatttggagtggtagtggttggtgttggcagagctggctcggtgaggctgagggacttgaag
K  R  K  F  G  V  V  V  V  G  V  G  R  A  G  S  V  R  L  R  D  L  K
gatccacgctctgcagcattcctgaacctgattggatttgtgtccagacgagagcttgggagccttgat
D  P  R  S  A  A  F  L  N  L  I  G  F  V  S  R  R  E  L  G  S  L  D
gaagtacggcagatttctttggaagatgctctccgaagccaagagattgatgtcgcctatatttgcagt
E  V  R  Q  I  S  L  E  D  A  L  R  S  Q  E  I  D  V  A  Y  I  C  S
gagagttccagccatgaagactatatacggcagtttctgcaggctggcaagcatgtcctcgtggaatac
E  S  S  S  H  E  D  Y  I  R  Q  F  L  Q  A  G  K  H  V  L  V  E  Y
cccatgacactgtcatttgcggcggcccaggagctgtgggagctggccgcacagaaaggagagtcctg
P  M  T  L  S  F  A  A  A  Q  E  L  W  E  L  A  A  Q  K  G  R  V  L
catgaggagcacgtggaactcttgatggaggaattcgaattcctgagaagagaagtgttggggaaagag
H  E  E  H  V  E  L  L  M  E  E  F  E  F  L  R  R  E  V  L  G  K  E
ctactgaaagggtctcttcgcttcacagctagcccactggaagaagagagatttggcttccctgcgttc
L  L  K  G  S  L  R  F  T  A  S  P  L  E  E  E  R  F  G  F  P  A  F
agcggcatttctcgcctgacctggctggtctccctcttcggggagctttctcttatttctgccaccttg
S  G  I  S  R  L  T  W  L  V  S  L  F  G  E  L  S  L  I  S  A  T  L
gaagagcgaaaagaggatcagtatatgaaaatgaccgtgcagctggagacccagaacaagggtctgctg
E  E  R  K  E  D  Q  Y  M  K  M  T  V  Q  L  E  T  Q  N  K  G  L  L
tcatggattgaagagaaagggcctggcttaaaaagaaacagatatgtaaacttccagttcacttctggg
S  W  I  E  E  K  G  P  G  L  K  R  N  R  Y  V  N  F  Q  F  T  S  G
tccctggaggaagtgccaagtgtaggggtcaataagaacattttcctgaaagatcaggatatatttgtt
S  L  E  E  V  P  S  V  G  V  N  K  N  I  F  L  K  D  Q  D  I  F  V
cagaagctcttaggccaggtctctgcagaggacctggctgctgagaagaagcgcatcatgcattgcctg
Q  K  L  L  G  Q  V  S  A  E  D  L  A  A  E  K  K  R  I  M  H  C  L
gggctggccagcgacatccagaagctttgccaccagaagaaggactacaaggatgacgatgacaagtag
G  L  A  S  D  I  Q  K  L  C  H  Q  K  K  D  Y  K  D  D  D  D  K  *
GGATATCTAGACCCAGCTT
```

Figure S2. Synthetic DNA sequence of *TP-BVRA*. Nucleotides with a gray background are adaptor sequences for cloning. The underlined lowercase sequence encodes the transit peptide of Rubisco small subunit 1A. The uppercase sequence is the linker region. The lowercase sequence encodes rat BVRA. The predicted amino acid sequence is given below the nucleotide sequence. The asterisk indicates the stop codon.

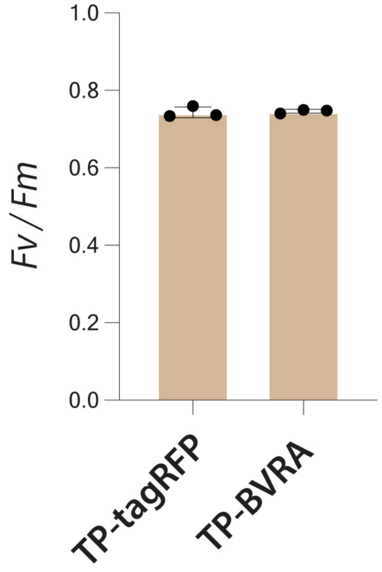

Figure S3. Measurement of F_v/F_m values in *N. benthamiana* leaves transiently expressing *TP-tagRFP* or *TP-BVRA*. Data are means ± SD (n = 3).

Table S1. Primers used in this study.

Name	Primer sequence (5´–3´)	Target
mCherry-F	ATGGTGAGCAAGGGCGAG	mCherry-UnaG
UnaGEcR	AAGCTGGGTCTAGATATCCTCATTCCGTCGCCCTCCG	TP-mCherry-UnaG / mCherry-UnaG
RBCS-SlF	AACCAATTCAGTCGACATGGCTTCCTCTATGCTC	Transit peptide of RBCS1A / ptUnaG / TP-UnaG-FLAG / TP-tagRFP
RBCS79mChe-R	GCCCTTGCTCACCATGGAATCGGTAAGGTCAGG	Transit peptide of RBCS1A / TP-UnaG / TP-tagRFP
UnaGfl-EcR	AAGCTGGGTCTAGATATCCCTTGTCATCGTCATCCTTGTAGTCTTCCGTCGCCCTCCGGTA	TP-UnaG-FLAG
RBCS79inv-F	GACCTTACCGATTCCGGAGGCTCAGGAGGCTCCATGGTCGAGAAATTT	TP-UnaG
RBCS79inv-R	AAATTTCTCGACCATGGAGCCTCCTGAGCCTCCGGAATCGGTAAGGTC	TP-UnaG
RBCS79RFP-R	CCTTAGACACCATGGAATCGGTAAGGTCAGGAAG	Transit peptide of RBCS1A
RBCS79RFP-F	CCTTACCGATTCCATGGTGTCTAAGGGCGAAG	tagRFP
RFPfl-EcR	AAGCTGGGTCTAGATATCCCTTGTCATCGTCATCCTTGTAGTCATTAAGTTTGTGCCCCAG	tagRFP / TP-tagRFP
NADK2-SlF	AACCAATTCAGTCGACATGTTCCTATGCTTTTGC	NADK2
NADK2-EcR	AAGCTGGGTCTAGATATCCGAGAGCCTTTTGATCAAG	NADK2
UnaG-attB1	GGGGACAAGTTTGTACAAAAAAGCAGGCTTCATGGTCGAGAAATTTGTT	Recombinant UnaG
UnaG-attB2	GGGGACCACTTTGTACAAGAAAGCTGGGTCTCATTCCGTCGCCCTCCGG	Recombinant UnaG
AtHO1-SlF	AACCAATTCAGTCGACTTAGTGGTGGTTGCGGCT	Recombinant Arabidopsis HEME OXGENASE 1
AtHO1-EcR	AAGCTGGGTCTAGATATCCTCAGGACAATATGAGACG	Recombinant Arabidopsis HEME OXGENASE 1
